# Determinants of delay in care seeking for diarrheal diseases among mothers/caregivers with under-five children in public health facilities of Arba Minch town, southern Ethiopia; 2019

**DOI:** 10.1371/journal.pone.0228558

**Published:** 2020-02-13

**Authors:** Alemayehu Fikire, Gistane Ayele, Desta Haftu

**Affiliations:** 1 Public Health Team, Hawassa College of Health Science, Hawassa, Ethiopia; 2 School of Public Health, Arba Minch University, Arba Minch, Ethiopia; Sefako Makgatho Health Sciences University, SOUTH AFRICA

## Abstract

**Background:**

Timely and appropriate health care seeking for diarrhea of under-five children is important to reduce severe and life-threatening complications. However, different findings indicate that mothers of under-five children often delay in seeking care which in turn contributes to the death of large number of children without ever reaching a health facility. Therefore, a proper pinpointing of determinants of delay in seeking care informs intervention strategies for health service planners.

**Objectives:**

Of this study was to identify the determinants of delay in care seeking for diarrheal disease among mothers/caregivers of under-five children in public health facilities of Arba Minch town, South Ethiopia, 2019.

**Methods:**

Facility based case control study was conducted from March 4 to April 30, 2019. Total sample size was 400. Cases were selected by systematic random sampling technique while controls were mothers of under-five children with signs and symptoms of diarrhea who came to the same health facility within 24 hours following cases. Data was collected by using pretested structured questionnaire by three data collectors and entered into EpiData V4 and exported to SPSS V23 for further analysis. Bivariable logistic regression was done to identify variables candidate for Multivariable LR at p-value<0.25. Multivariable logistic regression was done and p-value <0.05 and 95%CI of AOR was used to declare statistical significance.

**Result:**

Female sex[AOR = 1.93, (95%CI: 1.11,3.36)], child age <24 months[AOR = 4.47,95%CI:2.51,7.97)], mothers’/caregivers without formal education[AOR = 6.90, (95%CI:3.10,15.37)], and attended primary school [AOR = 3.12,(95%CI:1.44,6.73)], poorest household wealth index category[AOR = 2.81, (95%CI:1.20,6.58) and poor household wealth index category [AOR = 2.61,(95%CI: 1.12, 6.09)], mothers/caregivers who did not visit health facility to first episode diarrhea [AOR = 4.55, (95%CI:2.41,8.59)], mothers/caregivers who were satisfied in the last six month visit [AOR = 0.29, (95%CI:0.15,0.55)], and poor perceived health care professionals respect[AOR = 4.91, (95%CI:2.64,9.15)] were important determinants of delay in seeking care.

**Conclusions:**

Sex and age of the child, educational status of the mother/caregiver, poor wealth index category, not visiting health facility at first response, satisfaction with the care and examination, and respect of health care professionals were important determinants of delay in seeking care among mothers/caregivers of under-five children with diarrhea illness. All concerned body should focus interventions on poor and less educated mothers/caregivers with emphasis on female children and <24 months. Health workers are needed to provide respectful service to promote satisfaction level of clients.

## Background

Diarrhea is the passage of three or more loose or watery stools per 24 hours or an increase in stool frequency or liquidity that is mother of under-five child considered as abnormal[1, 2]. It is associated with many factors and causes fluid loss, in so doing contributes to the death of large number of children[3, 4]. Globally, it is one of the five leading causes of morbidity and mortality among under-five children. In 2017 alone, 5.4 million children died before reaching their fifth birthday, out of all deaths occurring among under-five children, Africa and South Asia jointly shared 78% in developing countries[5]. Moreover, Sub-Saharan Africa remains the region with the highest under-five mortality rate in the world, with 76 deaths per 1,000 live births[6]. In Ethiopia, 8% of under-five mortality was contributed by the diarrhea[4]. Seeking appropriate and prompt care could reduce by 20 percent of childhood deaths due to illnesses and prevent long-term complication[5]. However, health care seeking behavior of mothers of under-five children was poor in Ethiopia and only a small proportion of under-five children receive appropriate care timely [7–10]. Accordingly, EDHS 2016 revealed that only 44% of under-five children with diarrheal diseases were taken for advice or treatment to a health facility. Different studies also identified low health care seeking behaviour of mothers of under-five children with diarrheal diseases[8, 11–14]. In addition to that, even health care was sought, it was often delayed without ever reaching a health facility, and leads to morbidity and mortality of large number of under-five children, otherwise leads to long term complications[8, 11–14]. Ethiopian government has implemented integrated management of childhood illness (IMCI) protocols and community case management to improve access to care and health worker’s ability to treat under-five illnesses. In addition to that, Health Extension Workers provide community level disease prevention, health promotion and curative services. Moreover they were working to improve health care seeking behaviour of mothers/caregivers of under-five children with common childhood illness[15]. As a result, the health care seeking behaviour has showed progress and it has increased from 13% in 2000, 22% in 2005, 32% in 2011 and 44% in 2016[15, 16]. In addition to that; the country has successfully achieved some of the Millennium Development Goals (MDGs), like reduction of under-five mortality by more than two-thirds, three years in advance[5]. However, the health care seeking behaviour of mothers/caregivers of under-five children was low in general in the country as well as in the study area. For this reason, Identifying and having up-to-date evidence for the factors affecting the timely health care seeking behaviour is important for rational planning of intervention strategies, improve treatment compliance, evaluation of health care services and advance health promotion activities in a variety of contexts, particularly in Arba Minch town, south Ethiopia and the result may assist town and zonal health program planners, local NGOs and other bodies interested in reducing mortality and morbidity of under-five children. Therefore, this study identified determining factors of delay in care seeking for diarrheal diseases among mothers/caregivers of under-five children who visit public health facility of Arba Minch Town.

## Materials and methods

### Study area, study design and study population

An institution based case control study was conducted in public health facilities of Arba Minch town from March 4 to April 30, 2019. Arba Minch town is one of the city administrations found in Gamo Zone of SNNPR, Ethiopia. Arba Minch Town is located at a distance of 495 km south from Addis Ababa, capital of Ethiopia, and 275 km south west away from Hawassa, the regional capital. The town administration has an estimated total population of 115, 639 with male = 58,051, female = 57, 588, a total of 18,051under-five children in 2018/2019 G.C. It also has 1 general public hospital which is serving as referral hospital for the patients from surrounding districts in the zone as well as surrounding zones, and 2 public health centers[17]. Arba Minch General Hospital has emergency pediatric, OPD pediatric and inpatient pediatrics to provide health care service for children. Pediatric and child health OPD and wards has 4 pediatricians, 1from Hospital and 3 from Arba Minch University and 4 GP, 6 Health Officer, 5 BSc Nurses, 7 Clinical Nurses, 1 IMNCI trained professional. Arba Minch Health Center and Shecha Health Center have 2 and 1 IMNCI trained professionals, respectively who provide the care for under-five children. All mothers/caregivers of under-five children with diarrheal illness who utilize health care services from public health facilities of Arba Minch town were source population, and those mothers/caregivers of under-five children with diarrheal disease who were visited public health facilities during data collection period were study population. Cases were under-five children with signs/symptoms of diarrhea whose mothers sought treatment after 24-hours of the recognition of diarrhea[18, 19]. Controls were under-five children with signs/symptoms of diarrhea whose mothers sought treatment within 24-hours of the recognition of diarrhea[18, 19]. The inclusion criteria’s considered for the study subjects were all mothers/caregivers of under-five children seeking care for any type of diarrhea and visiting Pediatric/IMNCI clinic of public health facilities of Arba Minch town.

### Sample size determination and sampling technique

Sample size was determined by means of Epi Info Version 7 software by using sample size calculation formula for unmatched case control study. Sample size was calculated for maternal educational status, presence of severity signs and child’s age in months. Finally, we had taken child’s age in months as main exposure variable, with proportion of exposure among cases and controls was 74.1% and 60.1%, respectively, 80% of power, 95% confidence level, OR of 1.9, 1 to 1 case to control ratio, since it yields larger sample size, 380[18]. Considering 5% nonresponse rate, the final total sample size was 400.

All the public health facilities found in Arba Minch town were included in the study. Last year, 2010 EFY, same quarter 2-month report data was used to estimate number of participants from each health facility. Then, depending on that number, i.e. out of total 844 patients received care last year, sampling interval, K, was calculated, which was 2.11≈2. Proportional allocation of respondents to three public health facilities was done and the number of each case and control from Arba Minch General Hospital was 102, 55 from Arba Minch Health Center and 43 from Shecha Health Center. So, cases were selected by systematic random sampling technique where every other person was included and controls were patients who came to the same health facility following the cases. The first participant of the case group was selected by lottery method while the control was under-five child with diarrheal illness who came to the health facility following the cases but within the 24 hours of onset of diarrheal illness. When mothers/caregivers complained of diarrhea in their child completed their consultation with a health care professional, they were moved to a private room for an interview until the total required sample size obtained.

### Data collection procedure

Data was collected using semi-structured interviewer administered questionnaire which was adapted from EDHS_2016[20] and it includes predisposing, enabling, need/disease factors, promptness of treatment seeking, client perception towards pharmacy service, client satisfaction by the health facility service and client perception towards respect of health care professionals. Two diploma nurses in Heath Centers and 1 BSc Nurse in the Arba Minch General Hospital who were not assigned in under-five clinic to provide the service for children presenting with medical case were collected the data. Two BSc nurses supervised data collectors on daily bases to maintain the quality of data. Cases and controls were recruited from Hospital and health centers after they came to the facilities and are diagnosed with diarrhea and they were moved to private room to provide information.

### Data quality control

The questionnaire was initially prepared in English and it was translated into Amharic, local language, and back into English by language experts, to check the consistency. Two days training was given for data collectors and supervisors about the way of selecting study participants, way of interviewing and checking the already filled questionnaire by the principal investigator. Pretesting was performed at Shelle health center of Arba Minch Zuria district on 5% of sample size to check consistency and any ambiguity of the questionnaire a week back of actual data collection, then necessary revisions like some of the skipping pattern was modified. Data completeness was checked by the supervisor daily and weekly by the principal investigator for completeness and missing values.

### Data processing and analysis

Data was entered into EpiData Version 4.4.2.1 and exported to SPSS version 23 for further analysis. Data cleaning and coding was conducted. Descriptive analysis was carried out and summarized by frequency tables. Study participants’ household wealth status was assessed by principal component analysis. Then, participants were ranked into 5 groups, poorest, poor, middle, rich and richest based on the factor that explains largest variance in the included variables. The Hosmer Lemeshow statistics and deviance coefficient was used to check goodness of fit of the model and the model was good fit. No multicollinearity and outliers as it was checked by standard error of coefficients>2 and standardized residuals of >1.96 at 0.05 significance level respectively. Both bivariable and multivariable logistic regression was done to identify candidate variables at p-value <0.25 and determinants of delay in seeking timely care, respectively. Statistical significance of variables at final model was declared at p-value <0.05 and 95% CI of adjusted odds ratio.

### Operational definitions

#### Caregiver

Any person above 18 years of age who is directly responsible for the care of the child at the time of the study[14, 21].

#### Delay in seeking care

Care that was sought from health facilities after 24 hours from the recognition of the presence of diarrhea in under-five children[18].

#### Client perception towards pharmacy service

The perception of patients towards the pharmaceutical services provided in the health facility by pharmacy professionals were measured by 10 questions. The questions were organized to be responded in the likert scale, ranging from 1(strongly disagree) to 5(strongly agree). Then, the mean score was calculated, and the scores above the mean were considered to have positive perception towards the pharmacy service and those who score below the mean were considered to have negative perception toward the pharmacy services[22].

#### Client satisfaction

The satisfaction of patients with the service provided in the health facility by health professionals were measured with 12 questions. They were organized to be responded in the likert scale, ranging from 1(Poor) to 5(Excellent). The, the mean value was calculated and those who score above the mean were considered to have good satisfaction and those who score below the mean were considered to have poor satisfaction[22].

#### Client perception towards respect of health workers

The respect of health workers was measured in terms of patient’s response on the 5 questions provided. The questions were organized in likert scale, ranging from 1(strongly disagree) to 5(strongly agree). Then, the mean score was calculated and those who score above the mean were considered as they got respect from health workers and those who score below the mean were considered that as they did not get respect from health workers[23].

### Ethics and consent to participate

Ethical approval was obtained from the Institutional Review Board of Arba Minch University, College of Medicine and Health Science. A formal letter was written to Arba Minch Town health office and Arba Minch General Hospital for permission and support by Arba Minch University Department of Public Health. Then permission letter was written from Arba Minch Town health Unit to Health Centers. Informed verbal consent was obtained from actual study participants because the study is not sensitive, it has no physical harm and procedures. Health education on the effect of delayed care seeking was given on spot for those who came to health facility after 24 hours of recognizing symptoms of diarrhea. The aim of study was clearly explained to the mothers/caregivers of children of under-five years. The data was de-identified, de-linked and stored in a secure location. Respondents were assured about the confidentiality of the information they provided as well as their right to withdraw at any time during interview.

## Result

### Predisposing factors

In this study 400 mothers’/caregivers’ (200 cases and 200 controls) were interviewed, making the response rate of 100%. The majority,142(71.0%) of the cases were children <24 months but majority,121(60.5%) of controls were children ≥24 months. The age of cases ranges from 2 to 56 months, with a mean age of 22.24 (± 10.38SD) months and that of controls ranges from 2 to 58 months, with a mean age of 26.78 (± 12.22SD) months. More than half of respondents were females, 129(64.5%) among cases but it was males 110(55.0%) among controls (Table 1).

**Table 1:** Predisposing factors of delay in seeking care among mothers/caregivers of <5 children with diarrheal diseases in public health facilities of Arba Minch town, south Ethiopia, 2019 (n = 400).

Variables	Cases = 200		Controls = 200	
	No	%	No	%
Age of child in months				
<24	142	71	79	39.5
≥24	58	29	121	60.5
Sex of the child				
Male	71	35.5	110	55
Female	129	64.5	90	45
Birth order of the child				
≤2	120	60	132	66
3–4	76	38	65	32.5
≥5	4	2	3	1.5
Age category of the mother/caregiver				
15–25	94	47	67	33.5
26–34	81	40.5	79	39.5
≥35	25	12.5	54	27
Number of under-five children in the house hold				
1–2	83	41.5	94	47
3–4	74	37	69	34.5
> = 5	43	21.5	37	18.5
Place of residence				
Urban	146	73	150	75
Rural	54	27	50	25
Educational level of mother/caregiver				
No formal education	79	39.5	27	13.5
Primary education	50	25	44	22
Secondary education	44	22	60	30
College and above	27	13.5	69	34.5
Educational level of father				
No formal education	19	9.5	24	12
Primary education	58	29	36	18
Secondary education	84	42	72	36
College and above	39	19.5	68	34
Knowledge of diarrhea danger sign				
Poor	119	59.5	98	49
Good	81	40.5	102	51
Occupation of the mother/caregiver				
Housewife	109	54.5	90	45
Government employee	35	17.5	38	19
Merchant	38	19	46	23
Others	18	9	26	13
Occupation of the father				
Government employee	52	26	57	28.5
Merchant	44	22	58	29
Farmer	59	29.5	49	24.5
Daily laborer	38	19	31	15.5
Student	7	3.5	5	2.5
Mother/caregiver marital status				
Single	1	0.5	3	1.5
Married	195	97.5	189	94.5
Divorced	3	1.5	6	3
Widowed	1	0.5	2	1
Ethnicity of the mother/caregiver				
Gamo	117	58.5	119	59.5
Gofa	32	16	18	9
Amhara	27	13.5	32	16
Wolaita	21	10.5	27	13.5
Other*	3	1.5	4	2
Religion of the mother/caregiver				
Orthodox	87	43.5	89	49.5
Protestant	87	43.5	81	40.5
Catholic	10	5	16	8
Muslim	16	8	14	7

* indicates Guraghe, Oromo, Hadiya

### Enabling factors

Sixty-eight (34.0%) cases and 83(41.5%) controls home was at a distance of 15–30 minutes from preferred health facility on foot. Fifty (25.0%) cases belong to poorest wealth index, while 56(28.0%) controls were among richest wealth index group. More than half, 107(53.5%) cases and 103(51.5%) controls responded that the cost of treatment at the health facility was easy. Examination given, 85(42.5%) and short waiting time, 70(35.0%) among cases and short waiting time, 89(44.5%) among controls were the major reasons for the selection of health facility (Table 2).

**Table 2:** Enabling factors of delay in seeking care among mothers/caregivers of <5 children with diarrheal diseases in public health facilities of Arba Minch town, south Ethiopia, 2019(n = 400).

Variables	Cases = 200		Controls = 200	
	No	%	No	%
Wealth Index				
Poorest	50	25	25	12.5
Poor	43	21.5	32	16
Middle	29	14.5	47	23.5
Rich	39	19.5	40	20
Richest	39	19.5	56	28
Cost of treatment				
Easy to pay	107	53.5	103	51.5
Difficult to pay	52	26	61	30.5
Very difficult to pay	41	20.5	36	18
Distance to the nearest health facility in minutes				
<15 minutes	62	31	64	32
15–30 minutes	68	34	83	41.5
30–60 minutes	43	21.5	33	16.5
60–120 minutes	25	12.5	19	9.5
Above 120 minutes	2	1	1	0.5
Nearby health facility to the family				
Hospital	72	36	67	33.5
Health Centre	128	64	133	66.5
Preferred health facility				
Hospital	93	46.5	90	45
Health Center	107	53.5	110	55
Reason for preferring selected HF				
Do not charge too much	54	27	39	19.5
Nearness	71	35.5	75	37.5
Respect given	38	19	22	11
Examination given	85	42.5	69	34.5
Low waiting time	70	35	89	44.5
Treatment is effective	15	7.5	21	10.5
Always open	70	35	63	31.5
Necessary medication available	69	34.5	56	28

### Need/Disease related factors

Most of the mothers’/caregivers’112(56.0%) among cases, and 162(81.0%) among controls responded to diarrhea of children by taking them to the health facility at the first episode. About 65(32.5%) of cases and 47(23.5%) of controls took their children immediately when diarrhea of children was complicated with blood. But, only 35(17.5%) of cases and 53(26.5%) of controls mothers/caregivers reported that they seek medical care for any diarrhea. For the current diarrhea, 125(62.5%) of cases and 75(37.5%) of controls complained that child vomits everything eaten. Regarding type of diarrhea, 80(40.0%) of mothers/caregivers of case group and 89(44.5%) of mothers’/caregivers’ of control group complained watery type of diarrhea. The dehydration status of under-five children with diarrheal illness shows that 169(84.5%) and 167(83.5%) among cases and controls did not have dehydration respectively, some dehydration found to be 31(15.5%) and 33(16.5%) among cases and controls respectively, but no under-five child with severe dehydration among those who visited health facilities during study period (Table 3).

**Table 3:** Need/Disease related factors affecting delay in care seeking of mothers’/caregivers’ of <5 children with diarrheal diseases in public health facilities of Arba Minch town, south Ethiopia, 2019(n = 400).

Variables	Cases = 200		Controls = 200	
	No	%	No	%
Perceived severity of the illness				
Mild	121	60.5	98	49
Moderate	69	34.5	87	43.5
Sever	10	5	15	7.5
First response to child diarrhea				
Health Facility	112	56	162	81
Traditional, holy water treatment and buy drug from drug vendors	88	44	38	19
Symptoms of diarrhea at the time of seeking care at first response				
Blood in diarrhea	65	32.5	47	23.5
Child vomits everything	39	19.5	50	25
Child unable to feed/feed poorly	50	25	36	18
Eyeball sunken	11	5.5	14	7
For any diarrhea	35	17.5	53	26.5
Symptoms of current diarrhea and reason that initiated visit of health facility today				
Blood in diarrhea	62	31	39	19.5
Child vomits everything	125	62.5	75	37.5
Child unable to feed/feed poorly	97	48.5	63	31.5
Has fever	16	8	20	10
Sunken eyeball	63	31.5	33	16.5
Increased thirsty	9	4.5	11	5.5
Irritability/restlessness	5	2.5	20	10
Increased frequency of diarrhea	73	36.5	42	21
Only diarrhea	26	13	80	40
Husband or HEW told me to take to HF	4	2	9	4.5
No dehydration				
No	31	15.5	33	16.5
Yes	169	84.5	167	83.5
Some dehydration				
No	169	84.5	167	83.5
Yes	31	15.5	33	16.5
How your last 6 months’ visit helped for current visit				
Counseled about importance of early visit to health facility and danger of not visiting early	148	84.1	108	60
Satisfied with the respect, examination and care of child	28	5	72	26.5
Know child died of diarrhea				
Yes	14	7	11	5.5
No	186	93	189	94.5

### Patient perception towards health system related factors

Majority of respondents, 60.5% of case group have negative perception towards pharmacy service of the health facility, while about half of control group have such perception towards pharmacy service. Regarding client’s perception on respect of health care professional, more than half, 104(52.0%) among cases and 160(80.0%) among have good perception (Table 4).

**Table 4:** Patient perception towards health system related factors affecting delay in seeking health care from public health facilities of Arba Minch town, south Ethiopia, 2019.

	Case(n = 200)	Control(200)
	N(%)	N(%)
Patient perception toward pharmacy service		
Negative	121(60.5)	98(49.0)
Positive	79(39.5)	102(51.0)
Clients satisfaction with the health facility service		
Poor	19(9.5)	5(2.5)
Good	181(90.5)	195(97.5)
Clients perception on respect of health care professional		
Poor	96(48.0)	40(20.0)
Good	104(52.0)	160(80.0)

### Determinants of delay in care seeking

Bivariable and Multivariable binary logistic regression result was presented with 95% CI of both COR and AOR to identify candidate variables for multivariable logistic regression at p-value <0.25 and for determinants of delay in care seeking among mothers/caregivers of under-five children with diarrheal illness, respectively. Multivariable binary logistic regression result shows that sex and age of the child, mother/caregiver educational status, wealth index, mother/caregiver’s perception about the respect of health care professional, first response to child diarrhea, and how last visit helped were associated with delays in care seeking among mothers/caregivers of under-five children with diarrheal illness. Mothers/caregivers of female under-five children were 1.93 times (AOR = 1.93, 95%CI: 1.11, 3.36) more likely to delay than mothers/caregivers of male under-five children. The mothers/caregivers of young children (<24 months) were 4.47 times (AOR = 4.47, 95%CI: 2.51, 7.97) more likely to delay than mothers of older children (≥24 months). mothers/caregivers who did not attend formal education and attending primary education were 6.9 times (AOR = 6.90, 95%CI:3.10, 15.37) and 3.12 times (AOR = 3.12, 95%CI: 95%CI:1.44,6.73) more likely to delay than those who attended college and above education. Households wealth status was associated with delay in seeking care among mothers/caregivers of under-five children with diarrheal illness. The households belonging to poorest and poor wealth index category were 2.81 times (AOR = 2.81, 95%CI:1.20, 6.58) and 2.61 times (AOR = 2.61,95%CI:1.12, 6.09) more likely to delay when compared with richest wealth index category, respectively. Mothers/caregivers under-five children who did not visit health facility at first episode of diarrhea were 4.55 times (AOR = 4.55,95%CI:2.41, 8.59) more likely to delay when compared with those who had visited health facility. Mothers/caregivers who were satisfied with the care and examination of the child in the previous visit were 0.29 times less (AOR = 0.29,95%CI: 0.15–0.55) likely to delay than those who were only counseled on importance of early visit. Mothers/caregivers perception on the respect of health care professional was associated with the delay in seeking care. The mothers/caregivers who had poor perception on the respect of health care professional were 4.9 times (AOR = 4.91,95%CI:2.64, 9.15) more likely to delay than those who had good perception (Table 5).

**Table 5:** Multivariable logistic regression result on determinants of delay in care seeking among mothers/caregivers of <5-children with diarrheal illness in public health facilities of Arba Minch town, south Ethiopia, 2019 (n = 400).

Variables	Cases	Controls	COR (95% CI)	AOR(95%CI)
	(n = 200)	(n = 200)		
	No (%)	No (%)		
Child sex				
Female	129(64.5)	90(45.0)	2.22(1.49,3.32)*	1.93(1.11,3.36)**
Male	71(35.5)	110(55.0)	1	1
Child age in months				
<24	142(71)	79(39.5)	3.75(2.47,5.69)*	4.47(2.51,7.97)**
≥24	58(29)	121(60.5)	1	1
Educational status of the mother				
No formal education	79(39.5)	27(13.5)	7.48(4.01,13.95)*	6.90(3.10,15.37)**
Primary school	50(25.0)	44(22.0)	2.90(1.59, 5.30)*	3.12(1.44,6.73)**
Secondary school	44(22.0)	60(30.0)	1.87(1.04,3.38)*	2.11(.97,4.60)
College & above	27(13.5)	69(34.5)	1	1
Maternal/caregiver age				
15–24	94(46.0)	67(33.5)	3.03(1.72,5.35)*	
25–34	81(40.0)	79(39.5)	2.22(1.26,3.90)*	
35+	25(12.5)	54(27.0)	1	
Educational Status of the Father				
No formal education	19(9.5)	24(12.0)	1.38(0.67,2.83)*	
Primary school	58(29.0)	36(18.0)	2.81(1.54,4.98)*	
Secondary school	84(42.0)	72(36.0)	2.03(1.23,3.37)*	
College & above	39(19.5)	68(34.0)	1	
Media exposure on child issue (TV watch)				
NO	133(66.5)	92(46.0)	2.43(1.62,3.64)*	
Yes	67(33.5)	108(54.0)	1	
Wealth Index				
Poorest	50(25.0)	21(10.5)	3.75(1.97,7.15)*	2.81(1.20,6.58)**
Poor	43(21.5)	32(16.0)	2.12(1.16,3.88)*	2.61(1.12,6.09)**
Middle	24(12.0)	45(22.5)	0.84(0.44, 1.58)	1.06(0.46,2.45)
Rich	43(21.5)	39(19.5)	1.74(0.96,3.12)	0.97(0.41,2.30)
Richest	40(20.0)	63(31.5)	1	
Who decided first to take child to the HF				
Child’s father	77(38.5)	45(22.5)	2.14(1.35,3.41)*	
Grandparents	36(18.0)	46(23.0)	0.98(0.58,1.65)*	
Myself	87(43.5)	109(54.5)	1	
Clients Satisfaction by the health facility service				
Poor	19(9.5)	5(2.5)	4.09(1.49,11.19)*	
Good	181(90.5)	195(97.5)	1	
Client perception towards pharmacy service				
Negative	121(60.5)	98(49.0)	1.59(1.07,2.37)*	
Positive	79(39.5)	102(51.0)	1	
Client perception on respect of health professional				
Poor	96(48.0)	40(20.0)	3.69(2.37,5.75)*	4.91(2.64, 9.15)**
Good	104(52.0)	160(80.0)	1	1
First response to child diarrhea				
Visited other place than HF	88(44)	38(19)	3.35(2.14,5.26)*	4.55(2.41, 8.59)**
Visited HF	112(56)	162(81)	1	1
Knowledge of diarrhea danger signs				
Poor	119(59.0)	98(49.0)	1.53(1.03,2.27)*	
Good	81(40.5)	102(51.0)		
How last 6 months visit helped for today visit				
Counseled about importance of timely visit to HF	148(74)	108(54)	1	1
Satisfied with Rx, care and examination given	28(14.0)	72(36.0)	0.28 (0.17,0.47)*	0.29(0.15, 0.55)**

* Significant at p-value <0.25,

** significant at p-value <0.05

## Discussion

This research identified the predisposing, enabling, need/disease and health system related factors associated with delay in seeking health care for diarrheal illness and its overall result was in the expected direction and in line with most of the previous research findings in Jeldu, Woliso, Addis Ababa, Kersa district and Central Arsi Zone in Ethiopia, India, Rwanda and global trends report[7–9, 14, 18, 24–27]. Female children, children less than 24 months, mothers/caregivers without formal education and those attended only primary school, mothers/caregivers from poor wealth index households, Poor respondent’s perception on respect of health care professional, not visiting health facility as first response diarrhea, and mothers/caregivers who were satisfied with the care and examination of the child in the last six month were important predictors of delay in care seeking for diarrheal illness among mothers/caregivers of under-five children with diarrheal illness. Mothers/caregivers of female children were more likely to delay than mothers/caregivers of male children. The finding is consistent with previous research in Woliso, Ethiopia, India and rural Uganda[18, 24, 28] and study reported by Global DHS on care seeking and access to health care for childhood illness reported that males were more likely to seek care early[25]. In addition, the existing literature in different developing countries regarding health care seeking for females display that female disadvantage in health care seeking[29]. The possible reason for the difference in seeking care among mothers/caregivers of male and female children could be due to cultural influence, gender inequality that systematically disadvantage females in the community, which in turn may led to mothers/caregivers to pay attention only for males[29, 30]. This finding helps developing countries to plan intervention strategies including health education and communication on gender bias in seeking health care. However, this study is differed from studies conducted in rural Niger and Sierra Leone where gender had no significant association with either health care seeking and delay in seeking care[31, 32].

The mothers/caregivers of younger children (<24 months) were more likely to delay than those with older children (≥24 months). This finding is in line with previous studies conducted in Woliso of Ethiopia, Niger and India[18, 24, 25, 32]. The reason might be due to mothers/caregivers relate diarrhea with eruption of teeth that in younger children result in mild and self-limited diarrhea[18]. So, government and those who work on child health improvement programme should consider children age during planning intervention strategies. However, this finding is in contrary to the study from Malaysia and global reports on trend in care seeking and access of health service utilization, where young children were more likely to seek early care[25, 30]. In addition to this, the age of the child was not significant indicating that it has no any effect in decision of health care seeking process[10, 31].

The mothers/caregivers who did not attend formal education and attending primary education were more likely to delay than those who had college and above educational status. This pattern has been shown consistently in previous studies from Woliso and slums of Addis Ababa in Ethiopia, global trends report of 2015, Malaysia and India DHS data analysis report[7, 18, 24, 25, 30]. It is suggested that mothers/caregivers with no formal education or attended only primary school were less likely to recognize severe symptoms in their sick child, are more likely to seek traditional treatment and are less likely to have financial and other resources to take the sick child to the health facility, so, they are more likely to wait diarrhea improvement by itself at home. Moreover, mothers/caregivers with no formal education may not have basic awareness about the effect of delay in seeking care for children with diarrheal illness[12, 14, 33]. Therefore, intervention strategies should be designed according to the literacy level of mothers/caregivers of under-five children in order to have largest effect on child health improvement programme. However, this is in contrary to the EDHS 2011 and study conducted in rural Niger[10, 32]. This difference may be due to study design, study area and sample size, since EDHS 2011 data analysis used large sample.

The mothers/caregivers of poorest and poor wealth index category were more likely to delay than those who belong to richest wealth index category. This study finding is consistent with the study from slums of Addis Ababa in Ethiopia, EDHS 2011, Global trend in care seeking report, India DHS, Malaysia and seven rural districts of Sierra Leone [7, 10, 24, 25, 30, 31]. This may be due to a number of factors, like lack of money to pay user fee/medicine cost, transportation cost to and from health facility, and time lost from work/farming to take the child for treatment[10, 25]. Therefore, child health improvement programmers should design intervention strategies that encourage health insurance package in health service delivery and free service for under-five children in public health facilities. However, it was in contrary to the study from Woliso of Central Ethiopia, Jeldu district of Ethiopia and Kigali in Rwanda, where monthly income/wealth index of respondents had no significant association with health care seeking behavior [14, 18, 27]. This might be due to that respondents who have been utilizing these health facilities may have little variation in their capacity to pay service user fee or medicine cost in these study areas.

Visiting health facility at first episode of childhood diarrhea was associated with early care seeking of mothers. The mothers/caregivers of under-five children who did not visit health facility as first response to diarrhea were more likely to delay than those who had visited health facility. This study finding was consistent with the study conducted in Woliso of central Ethiopia and Kersa district of eastern Ethiopia[9, 18]. This may be associated with the counseling from health care professional providing the service at the first episode of diarrhea on early visit to or the client may be satisfied with the service provided in the health facility[18]. This finding indicates that providing health education on timely visit to health facility improves health care seeking behaviour of mothers/caregivers of under-five children. Therefore, intervention strategies should consider providing health education on the importance of early health care seeking for the community at the community as well as health facility level. However, this finding is contrary to study conducted in Rwanda, where mothers/caregiver of under-five children with diarrheal illness who had first consultation at public health institution were more likely to be delayed[27]. This may be due to mothers/caregivers of under-five children may be dissatisfied with the service provided and may wait at home or take the sick child to traditional treatment, so they may take their sick child after complications has developed.

The mothers/caregivers who were satisfied with the care and examination of the child in the previous visit were less likely to delay than those who were only counseled on importance of early visit. This study finding is consistent with the study conducted in Woliso of Central Ethiopia and evidence based national survey of Ethiopia[18, 34]. Health facility managers as well as health care professionals should focus on providing quality service with respect to improve satisfaction of mothers/caregivers of under-five children in order to have largest effect on child health improvement programme.

Health care professionals respect to clients was also important determinant of delay in seeking health care in this study. The mothers/caregivers who have poor perception on the respect of health care professional were more likely to delay than those with good perception. However, the effect of this variable was not studied previously.

Cost of treatment and health facility distance were not predictors of delay among mothers/caregivers of under-five children with diarrheal illness. This is contrary to other studies that cited cost and distance from nearby health facility as an important determinant for delay in seeking and not seeking health care. However, different studies in Ethiopia and Niger support this finding. The difference may be due to the fact that capacity to pay for service fee or medicine cost at health facilities may not vary to a large extent, so that most of them report that the cost is easy to pay (15,23). In addition, this study was conducted in town administration where most of the study participants were from the town, distance is less likely to be a barrier for early seeking health care.

## Conclusion

This study revealed that child’s age in months, child sex, educational status of the mother/caregiver, visiting health facility as first response to childhood diarrhea, wealth index category of the household, health care professionals respect to the clients during service provision were important determinants of delay in health care seeking among mothers/caregivers of under-five children. On the other hand, satisfaction with care and examination of the child in the last six month visit to the health facility has protective effect on the delay in seeking health care among mothers/caregivers of under-five children with diarrheal illness. Therefore, consider intervention strategies including health education and communication on misinterpretation of diarrheal disease among children <24 months and cultural beliefs that link diarrhea with eruption of milk teeth during health care seeking with emphasis on poor and less educated families, families with female under-five children to have largest impact on child health improvement programme. Focus on providing quality health care services that satisfy mothers of under-five children, create awareness on effect of delayed care seeking and diarrhea danger signs among mothers/caregivers of under-five children should also be well thought-out.

## Supporting information

S1 File English version questionnaire used to collect data at southern Ethiopia, 2019. (DOCX)

S2 File Amharic version questionnaire used to collect data at southern Ethiopia, 2019. (DOCX)

S3 File SPSS final data for southern Ethiopia. (SAV)

## Abbreviations

ADD: Acute Diarrheal Disease
AOR: Adjusted Odds Ratio
CI: Confidence Interval
CUFY: Child Under-five years
EDHS: Ethiopian Demographic and Health Survey
EFY: Ethiopian Fiscal Year
FMOH: Federal Ministry of Health
HCSB: Health Care Seeking Behavior
LMIC: Low- and Middle-Income Countries
NGO: Nongovernmental Organization
OR: Odds Ratio
