# Supplementary material for: Determinants of delay in care seeking for diarrheal diseases among mothers/caregivers with under-five children in public health facilities of Arba Minch town, southern Ethiopia; 2019
Source: PLoS One. 2020 Feb 13;15(2):e0228558. doi: 10.1371/journal.pone.0228558 (PMC7018063; doi:10.1371/journal.pone.0228558)
Supplement: S1 File — (DOCX) [file pone.0228558.s001.docx]

English Version Questionnaire to interview study participants.

Questionnaire Code: ___________

**Instruction:** choose the appropriate answers of the study participants for each of the following questions.

| **Section 1. General Information about the child and parental socio-demographic characteristics** | | | |
| --- | --- | --- | --- |
| No | Questions | Coding categories | Skip |
| 101 | Child’s age | _________(in months) |  |
| 102 | Age of mother/caregiver | _________(in years) |  |
| 103 | Sex of the child | 1. Male 2. Female |  |
| 104 | Birth order of the child | 1. First 2. Second 3. Third 4. Fourth 5. Fifth and above |  |
| 105 | Number of under-five children in the household | _____________(in number) |  |
| 106 | Place of residence | 1. Rural 2. Urban |  |
| 107 | Marital Status of mother/caregiver | 1. Single 2. Married 3. Divorced 4. Widowed |  |
| 108 | Religion of the mother/caregiver | 1. Protestant 2. Orthodox 3. Catholic 4. Muslim |  |
| 109 | Ethnicity | 1. Gamo 2. Gofa 3. Amhara 4. Wolaita 5. Others, Specify___ |  |
| 110 | Educational Status of mother/caregiver | 1. No formal education 2. Primary education 3. Secondary education 4. Certficate 5. Diploma and above |  |
| 111 | Do you involve in community conversation programme? | 1. Yes 2. NO |  |
| 112 | Do you watch TV or Listen to radio about issues of the children? | 1. Yes 2. NO |  |
| 113 | Educational level of father | 1. No formal education 2. Primary education 3. Secondary education 4. Certificate 5. Diploma and above |  |
| 114 | Mother/caregivers occupation | 1. House wife 2. Gov’t employee 3. Merchant 4. Farmer 5. Labor worker 6. Student 7. Other specify_______ |  |
| 115 | Occupation of father | 1. Gov’t employee 2. Merchant 3. Farmer 4. Labor worker 5. Student 6. Other specify_______ |  |
| 116 | Number of family members | ________________in Numbers |  |
| 117 | Do you know severity/danger signs of diarrhea? | 1. Yes 2. No |  |
| 118 | If yes to Q.116, then what are them? | 1. Sunken eyeball  2. Lethargic  3. Not able to drink/drinking too  poorly  4. Skin pinch goes back very slowly  5. Restless/irritable |  |
| **Section 2: Questions that assess enabling factors** | | | |
| **Wealth Index variables** | | | Number |

| 201 | Do have your own home? | 1. Yes 2. No |  |
| --- | --- | --- | --- |
| 202 | Does your household have the following properties? | |  |
|  | Electricity? | 1. Yes 2. No |  |
|  | Radio? | 1. Yes 2. No |  |
|  | Television? | 1. Yes 2. No |  |
|  | Non-mobile telephone? | 1. Yes 2. No |  |
|  | Computer? | 1. Yes 2. No |  |
|  | Refrigerator? | 1. Yes 2. No |  |
|  | Table? | 1. Yes 2. No |  |
|  | Chair? | 1. Yes 2. No |  |
|  | Bed with cotton sponges/ spring mattress? | 1.Yes 2. No |  |
|  | Electric mitad? | 1.Yes 2. No |  |
|  | Kerosene lamp/pressure lamp? | 1. Yes 2. No |  |
| 203 | Does any member of your household own the following? | | |
|  | Mobile phone? | 1.Yes 2. No |  |
|  | Bicycle? | 1. Yes 2. No |  |
|  | Motorcycle? | 1. Yes 2. No |  |
|  | Animal-drawn cart? | 1.Yes 2. No |  |
|  | Car? | 1.Yes 2. No |  |
|  | Baggage? | 1. Yes 2. No |  |
| 204 | Does any one of your household have bank account? | 1. Yes 2. No |  |

| 205 | What is the cost of treatment in health facility for child hood diarrhea? | 1. Easy to pay 2. Difficult to pay 3. Very difficult to pay |  |
| --- | --- | --- | --- |
| 206 | Are you the member of community based health insurance(CBHI) currently? | 1. Yes 2. No |  |
| 207 | Which health facility is close to your home? | 1. Government 2. Private |  |
| 208 | Which health facility do you prefer for children with diarrhea? | 1. Government 2. Private |  |
| 209 | Why did you prefer the selected health facility?  **(Circle all responses)** | 1. They do not charge to much 2. Because, they are near 3. Because, they are respectful 4. Thorough examination 5. Low waiting time 6. Necessary medication is available 7. Treatment is effective 8. Always open or early opening 9. Other (Specify)…… |  |
| 210 | How close is the selected facility on foot? | 1. <15 minutes 2. 15-30 minutes 3. 30-60 minutes 4. One-Two hour 5. Abovetwo hours |  |
| **Section 3: Questions that assess need/disease related factors** | | | |
| 301 | What was your first response when your child had diarrhea? | 1. Take to health facilities 2. Take to traditional treatment 3. Self-treatment at home 4. Treat the child with drug buying from pharmacies or drug sellers without prescriptions 5. Treat with holy water 6. Did nothing |  |
| 302 | When did you seek medical care for diarrhea of children? | 1. Blood in diarrhea 2. Child vomits every thing 3. Child unable to feed or feed poorly 4. Has fever 5. Eye ball sunken 6. For any diarrhea 7. Other (specify)…… |  |
| 303 | What make you seek medical care  today? | 1. Blood in diarrhea 2. Child vomits every thing 3. Child unable to feed or feed poorly 4. Has fever 5. Eye ball sunken 6. Increased thirsty 7. Irritability or restlessness 8. Increased frequency of diarrhea 9. Only diarrhea 10. Someone (Husband, HEW, neighbor) told to take the child to health facility 11. Other (specify)…… |  |
| 304 | How many times your child passes the stool per day? (day and night) | ____________no of diarrhea |  |
| 305 | What is the type of diarrhea? | 1. Bloody 2. Mucoid 3. Watery 4. Other specify________ |  |
| 306 | Who decided first to take the child for medical treatment? | 1. My self 2. Father 3. Grand parents 4. Other specify_______ |  |
| 307 | Did the child encounter diarrhea  before in the last six months? | 1. Yes 2. NO |  |
| 308 | If yes to Q307, did you visit health  facility? | 1. Yes 2. NO | **If No, skip to Q311** |
| 309 | If yes to Q308, did that visit help  you for your today’s visit? | 1. Yes 2. NO |  |
| 310 | If Yes to Q309, how? | 1. Counseled about the importance of visiting health facility for diarrhea 2. Told the danger of not visiting health facility for diarrhea 3. Satisfied with the treatment given 4. Satisfied with the respect given 5. Satisfied with examination of child 6. Other (specify)……………. |  |
| 311 | If No to Q308, how was the child  cured? | 1. Take to traditional treatment 2. Self-treatment at home 3. Treat the child with drug buying from pharmacies or drug sellers without Prescriptions 4. Treat with holy water 5. Resolved by it self 6. Other specify_______ |  |
| 312 | Is there a child died of diarrhea in the family or neighbor before? | 1. Yes 2. NO |  |
| 313 | Do you know the cause of diarrhea? | 1. Drinking contaminated water 2. Poor hygiene and sanitation 3. Eating contaminated food 4. Teething 5. Evil eye 6. Infection/weaning 7. Others, specify |  |
| 314 | What is the dehydration status of the child? (**check from the record**) | 1. No dehydration 2. Some dehydration 3. Severe dehydration |  |
| **Section 4: Questions that assess promptness of treatment seeking for diarrhea in children** | | | |
| 401 | How long your child had illness  before seeking medical care? | 1. within < 24 hours after the onset of diarrhea 2. After 24-48 hours of onset of diarrhea 3. After 48-72 hours of onset of diarrhea 4. After 3-7 of onset of diarrhea 5. After seven days of onset of diarrhea | **Skip if within one day to Q405** |
| 402 | If after one day of onset of diarrhea, what were the main reasons you were not seek medical care immediately? | 1. The disease resolves by itself over time 2. The disease resolved by itself before on the child or other child 3. Transportation difficulties. 4. Cost of medical care 5. No trust on health providers’ competency. 6. Fear that S/he don't get immediate care or several waiting time in facility 7. Giving traditional medication at home 8. Buy medication from drug vendors 9. No treatment for the sickness 10. Lack of time 11. Shortage/lack of money 12. Illness was mild 13. Other (specify)……….. |  |
| 403 | If you select traditional healer, what is the most important reason for choosing traditional healers | 1. Don’t get cure from medical care 2. They do not charge to much 3. They are respectful 4. There is no long waiting time 5. Treatment is effective 6. Maintain confidentiality 7. Maintain privacy 8. Because, family recommended it 9. Because, they are near 10. Other (specify)……. |  |
| 404 | If within one day of the onset of diarrhea, what makes you urge to  seek medical care early? | 1. Previous experience of the delay result in severity 2. Symptoms worsen 3. Get the information about importance of early seeking for diarrhea. 4. Other (specify)…… |  |
| 405 | If you get information about the importance of early treatment seeking for childhood diarrhea, what is your source of information? | 1. Health workers 2. HEWs through training or health education 3. During community conversation 4. Neighbors 5. Media (Television, radio) 6. Others specify_______ |  |

| **Part V:-Questions that assess client’s perception toward pharmaceutical services** | | | | | | |
| --- | --- | --- | --- | --- | --- | --- |
|  | | Strongly agree | Agree | Neutral | Disagree | Strongly disagree |
| 501 | Pharmacy services are essential for health care provision during the illness | 🞏 | 🞏 | 🞏 | 🞏 | 🞏 |
| 502 | Pharmacists counsel you about the directions for use of medications | 🞏 | 🞏 | 🞏 | 🞏 | 🞏 |
| 503 | Pharmacists can counsel and educate you on your medication and drug therapy uses | 🞏 | 🞏 | 🞏 | 🞏 | 🞏 |
| 504 | Pharmacists can intervene you in the event of any form of problem with your treatment | 🞏 | 🞏 | 🞏 | 🞏 | 🞏 |
| 505 | Pharmacists have responsibilities to monitor your response to drugs | 🞏 | 🞏 | 🞏 | 🞏 | 🞏 |
| 506 | Pharmacists are responsible for providing you medications in the hospital pharmacies | 🞏 | 🞏 | 🞏 | 🞏 | 🞏 |
| 507 | Pharmacists are very helpful in drug product selection | 🞏 | 🞏 | 🞏 | 🞏 | 🞏 |
| 508 | Pharmacists are not competent to give advice on health matters | 🞏 | 🞏 | 🞏 | 🞏 | 🞏 |
| 509 | Pharmacists are health care providers | 🞏 | 🞏 | 🞏 | 🞏 | 🞏 |
| 510 | You can discuss very serious health problems with pharmacists | 🞏 | 🞏 | 🞏 | 🞏 | 🞏 |
| **Part VI: -Questions used to assess client’s satisfaction with the health facility services** | | | | | | |
|  | | Excellent | V.Good | Good | Fair | Poor |
| 601 | The availability of medicines that are prescribed to you in the pharmacy | 🞏 | 🞏 | 🞏 | 🞏 | 🞏 |
| 602 | The care that the health professional takes while providing care | 🞏 | 🞏 | 🞏 | 🞏 | 🞏 |
| 603 | The privacy of your conversations with the Doctor/health worker | 🞏 | 🞏 | 🞏 | 🞏 | 🞏 |
| 604 | How well the doctor/health worker explains possible side effects | 🞏 | 🞏 | 🞏 | 🞏 | 🞏 |
| 605 | The fairness of cost of medicines in the pharmacy | 🞏 | 🞏 | 🞏 | 🞏 | 🞏 |
| 606 | The amount of time the Doctor and pharmacy professional spends with you | 🞏 | 🞏 | 🞏 | 🞏 | 🞏 |
| 607 | The clarity of the pharmacy professionals’ instructions about how to take your medication | 🞏 | 🞏 | 🞏 | 🞏 | 🞏 |
| 608 | Way questions & queries dealt by staff | 🞏 | 🞏 | 🞏 | 🞏 | 🞏 |
| 609 | Overall waiting time | 🞏 | 🞏 | 🞏 | 🞏 | 🞏 |
| 610 | The amount of time you spend waiting for your prescription to be filled | 🞏 | 🞏 | 🞏 | 🞏 | 🞏 |
| 611 | Access and cleanliness of latrines | 🞏 | 🞏 | 🞏 | 🞏 | 🞏 |
| 612 | Overall level of satisfaction | 🞏 | 🞏 | 🞏 | 🞏 | 🞏 |
| **Part VII: -Patient waiting time** | | | | | | |
| 701 | How long did you stay before leaving the facility after the service in the last 6 months visit | ________________(minutes) | | | | |
| **Part VIII: - Questions that assess respect of health care professional** | | | | | | |
|  |  | Strongly agree | Agree | Neutral | Disagree | Strongly disagree |
| 801 | Talked with me in a friendly way | 🞏 | 🞏 | 🞏 | 🞏 | 🞏 |
| 802 | Asked me whether I want my family to take part in my care. | 🞏 | 🞏 | 🞏 | 🞏 | 🞏 |
| 803 | Listened to my personal wishes with regard to my care. | 🞏 | 🞏 | 🞏 | 🞏 | 🞏 |
| 804 | Helped me take part in decisions concerning my care. | 🞏 | 🞏 | 🞏 | 🞏 | 🞏 |
| 805 | Helped me express my opinions on my care. | 🞏 | 🞏 | 🞏 | 🞏 | 🞏 |
