# Supplementary material for: Determinants of delay in care seeking for diarrheal diseases among mothers/caregivers with under-five children in public health facilities of Arba Minch town, southern Ethiopia; 2019
Source: PLoS One. 2020 Feb 13;15(2):e0228558. doi: 10.1371/journal.pone.0228558 (PMC7018063; doi:10.1371/journal.pone.0228558)
Supplement: S2 File — (DOCX) [file pone.0228558.s002.docx]

የቃለ መጠይቁ ኮድ፡_________________የጤና ተቋሙ ስም__________________________

**የቃለመጠይቁ ትዕዛዝ፡** የጥናቱ ተሳታፍ የምመልሰዉን መልስ ብቻ ክበብ፡፡

| **ክፍል አንድ፡ -አጋላጭ/የስነ ማህበራዊና ስነ እኮኖምያዊ መረጃዎች፡፡** | | | | ዝለል |
| --- | --- | --- | --- | --- |
| 101 | የህጻኑ እድሜ? | _________________በወር | |  |
| 102 | የእናት/ተንከባካብ እድሜ? | _________________በዓመት | |  |
| 103 | የህጻኑ ጾታ? | 1. ወንድ  2. ሴት | |  |
| 104 | ህጻኑ ከእናቱ ልጆች ስንተኛ ስለመሆኑ? | 1. አንደኛ  2. ሁለተኛ  3. ሦስተኛ  4. አራተኛ | |  |
| 105 | የመኖርያ አድራሻ? | 1. ገጠር  2. ከተማ | |  |
| 106 | የእናቱ የጋብቻ ሁኔታ? | 1. ያላገባች  2. ያገባች  3. የተፋታች  4. ባል የሞተባት | |  |
| 107 | የእናቱ/ተንከባካብዉ/ዋ ብሄረሰብ? | 1. ጋሞ  2. ጎፋ  3. አማራ  4. ወላይታ  5. ሌላ ካለ ይጥቀሱ | |  |
| 108 | የእናት/ተንከባካብዉ/ዋ ሐይማኖት? | 1. ኦርቶዶክስ  2. ፕሮተስታንት  3. ካቶልክ  4. ሙስሊም  5. ሌላ ካለ ይጥቀሱ | |  |
| 109 | የእናት/የተንከባካብዉ/ዋ ት/ት ደረጃ? | 1. መደበኛ ት/ት ያልተማረች  2. የመጀመርያ ደረጃ ት/ት የጨረሰች  3. የሁለተኛ ደረጃ ት/ት የጨረሰች  4. ኮሌጅና ከዝያ በላይ | |  |
| 110 | የእናት/የተንከባካብዉ/ዋ ሥራ ዓይነት? | 1. የቤት እሜቤት  2. የመንግስት ተቀጣር  3. ነጋዴ  4. አርሶ አደር  5. የቀን ሠራተኛ  6. ተማር  7. ሌላ ካሌ ይጥቀሱ | |  |
| 111 | የህጻኑ አባት የት/ት ደረጃ? | 1. መደበኛ ት/ት ያልተማሬ  2. አንደኛ ደረጃ ያጠናቀቀ  3. ሁለተኛ ደረጃ ያጠናቀቀ  4. ሠርትፍኬት ያለዉ  5. ዲፒሎማና ከዝያ በላይ | |  |
| 112 | የህጻኑ አባት ሥራ ዓይነት? | 1. የመንግስት ተቀጣር  2. ነጋዴ  3. አርሶ አደር  4. የቀን ሠራተኛ  5. ተማር  6. ሌላ ካሌ ይጥቀሱ | |  |
| 113 | የቤተሰብ ብዛት ስንት እንደሆኔ? | ______________(በቁጥር) | |  |
| 114 | የተቅማጥ በሽታ አደገኛ ምልክቶችን ያዉቃሉ | 1. አዎ  2. አላዉቅም | **If 2, skip to 116** | |
| 115 | ለጥያቄ ቁጥር 116 አዎ ከሆነ፤ ምን ምን ናቸዉ? | 1. ወደ ዉስጥ የሰረጎዴ አይን  2. ህጻኑን በጣም ከደከመዉ  3. መጠጣት የማይችል/በትንሹ ብቻ የምጠጣ  4. ቆዳዉ ከተያዘበት ስለቀቅ በጣም ቀስ ብሎ የምመለስ ከሆነ  5. ህጻኑ እረፍት የምያጣ/የምበሳጭ ከሆነ | |  |
| 116 | በጤና ኤክስተንሽን ፓኬጅ ተመርቀሽ ነበር? | 1. አዎ  2. አይደለም | |  |
| 117 | በማህበረሰብ አቀፍ ዉይይት ተሳትፈሽ ታዉቅ ነበር? | 1. አዎ  2. አላዉቅም | |  |
| 118 | ስለህጻናት ጤና ጉዳይ የምያስተምረዉን የተለቨዥን ዎይም የሬድዮ ፕሮገራም ተከታትለዉ ያዉቃሉ? | 1. አዎ  2. አላዉቅም | |  |
| **ክፍል ሁለት፡ -አመቻች ሁኔታዎች** | | | | |
| 201 | አማካይ የወር ገብያችሁ ምን ያህል ይሆናል | _____________ብር/በወር | |  |

| 202 | የግል መኖርያ ቤት አላችሁ? | 1. አዎ  2. የለንም | |  |
| --- | --- | --- | --- | --- |
| 203 | ለቤተሰባችሁ የሚከተሉት ካላችሁ አዎ ዎይም አይደለም ብለዉ ይመልሱ | | ካሉ ቁጥር ይጥቀሱ | |
|  | መብራት አላችሁ | 1. አዎ  2. አይደለም | |  |
|  | ሬድዮ አላችሁ? | 1. አዎ  2. አይደለም | |  |
|  | ቴሌቪዥን አላችሁ? | 1. አዎ  2. አይደለም | |  |
|  | የቤት ስልክ አላችሁ? | 1. አዎ  2. አይደለም | |  |
|  | ኮፒዉቴር አላችሁ? | 1. አዎ  2. አይደለም | |  |
|  | ፍርጅ/የዉሃ ማቀዚቀዣ አላችሁ | 1. አዎ  2. አይደለም | |  |
|  | ጠረጴዛ አላችሁ? | 1. አዎ  2. አይደለም | |  |
|  | ወንበር አላችሁ? | 1. አዎ  2. አይደለም | |  |
|  | የጥጥ(ስፓንጅ) ስፕሪንግ ፍራሽ ያለው አልጋ አላችሁ? | 1. አዎ  2. አይደለም | |  |
|  | የመብራት ሚጣድ አላችሁ? | 1. አዎ  2. አይደለም | |  |
| 204 | ማንኛዉም የዚህ ቤተሰብ አባል የሚከተሉት ካላችሁ አዎ ዎይም አይደለም ብለዉ ይመልሱ | | | |
|  | የግርግዳ ሰዓት አላችሁ? | 1. አዎ  2. አይደለም | |  |
|  | ተንቀሳቃሽ ስልክ? | 1. አዎ  2. አይደለም | |  |
|  | ብስክለት አላችሁ? | 1. አዎ  2. አይደለም | |  |
|  | ሞተር ሳይክል አላችሁ? | 1. አዎ  2. አይደለም | |  |
|  | የእንስሳት ጋሪ አላችሁ? | 1. አዎ  2. አይደለም | |  |
|  | የሞተር ጀልባ አላችሁ? | 1. አዎ  2. አይደለም | |  |
|  | ባጃጅ አላችሁ? | 1. አዎ  2. አይደለም | |  |
|  | መኪና አላችሁ? | 1. አዎ  2. አይደለም | |  |
| 205 | ማንኛዉም የእናንተ ቤተሰብ አባላት የባንክ ቤት ቡክ አላችሁ? | 1. አዎ  2. አይደለም | |  |

| 206 | የዝህ ጤና ተቋም የተቅማጥ ህክምና ወጭን እንዴት ይምዝናሉ? | 1. ለመክፌል ቀላል ነዉ  2. ለመክፌል ከባድ ነዉ  3. ለመክፌል በጣም ከባድ ነዉ | | | |  |  |
| --- | --- | --- | --- | --- | --- | --- | --- |
| 207 | ለቤታችሁ የምቀርብ ጤና ተቋም ዬቱ ነዉ? | 1. ሆስፕታል  2. ጤና ጣብያ | | | |  |  |
| 208 | በተቅማጥ የታመመ ህጻን ለማሳከም ዬትኛዉን ጤና ተቋም ነዉ የምትመርጨዉ? | 1. ሆስፕታል  2. ጤና ጣብያ | | | |  |  |
| 209 | ለምን ነበር ያንን ጤና ተቋም የመረጥሽዉ?  (ሁሉንም መልስ ክቤብ) | 1. ብዙ ብር ስለማይጠይቁ  2. ቅርብ ስለሆነ  3. ተገልጋይ አክባር ስለሆኑ  4. በደንብ ምርመራ ስለምያደርጉ  5. ብዙ ስለማያስጠብቁ  6. አስፈላግ መድኃኒት ሁሉ ስላላቸዉ  7. ህክምናቸዉ ወድያዉ የምፈዉስ ስለሆነ  8. ሁለ ሰለምከፈት/በግዜ ስለምከፈት  9. ሌላ ካለ ይጥቀሱ | | | |  |  |
| 210 | የመረጥሽዉ ጤና ተቋም በእግር ምን ያህል ሳዓት ያስከዳል? | 1. ከ15 ደቅቃ በታች  2. ከ15-30 ደቅቃ  3. ከ30-60 ደቅቃ  4. ከ1-2 ሳዓት  5. ከ2 ሳዓት በላይ | | | |  |  |
| **ክፍል ሶስት፡ -ከበሽታ ጋር የተያያዙ ምክንያቶች** | | | | | | | |
| 301 | ከዝህ በፊት ለመጀመርያ ግዜ ህጻንሽ በተቅማጥ ስያዝ ምን ነበር ያደረግሽዉ? | 1. ጤና ተቋም ወስጀያለሁ  2. ለባህላዊ ህክምና ወስጀዋለሁ  3. እነ እራሰ በቤተ አክመዋለሁ  4. ከመድሐኒት መደብር/ከሱቅ መድሓኒት ገዝቸ አክመያለሁ  5. በቅዱስ ዉሓ አክመዋለሁ  6. ምንም አልተደረገም | | | |  |  |
| 302 | ከዝህ በፊት መቸ ነበር ለህጻንሽ ተቅማጥ ህክምና አገልግሎት የፈለግሽዉ? | 1. በደም የተቀላቀለ ተቅማጥ ስሆን  2. የበላዉን ሁሉ የምያስመልሰዉ ከሆኔ  3. ህጻኑ ምብላት ስያቅተዉ/ትንሽ ትንሽ የምበላ ከሆኔ  4. ትኩሳት ስኖረዉ  5. አይኑ ወደ ዉስጥ ስለሰረጎዴ  6. ለሁሉም ዓይነት ተቅማጥ  7. ሌላ ካለ ይጥቀሱ | | | |  |  |
| 303 | ዛሬ የህክምና አገልግሎት እንድትፌልግ ያደረገሽ ምን ነበር? | 1. በደም የተቀላቀለ ተቅማጥ ስለሆነ  2. ህጻኑን ሁሉንም ነገር ስለምያስታዉከዉ  3. ህጻኑ መብላት ስላቃተዉ/በቂ ምግብ  መመገብ ስለማይችል  4. ትኩሳት ስላለዉ  5. ዓይኑ ወደ ዉስጥ መሰርጎዱ  6. ከበፊቱ ይልቅ ስለምጠማዉ  7. ስለምጨናነቅ/ስለማያርፍ  8. የምያስቀምጥበት ቁጥር ስለጨመሬ  9. ተቅማጥ ብቻ ስለነበረዉ  10. ሰዉ(ባለ፤ጤ/ኤ/ሠ፤ጎሮቤት) ጤና ተቋም እንድወስድ ስለነገረኝ  11. ሌላ ካለ ይጥቀሱ | | | |  |  |
| 304 | ህጻንሽን በቀን ስንት ግዜ ነበር ስያስቀምጥ የነበረዉ(በቀንና በሌልት)? | __________ (ተቅማጥ በቁጥር) | | | |  |  |
| 305 | ተቅማጡ ምን ዓይነት ነበር? | 1. በደም የተቀላቀለ  2. ንፍጥ የተቀላቀለ  3. ዉሃማ  4. ሌላ ካሌ ይጥቀሱ | | | |  |  |
| 306 | ህጻኑን ለህክምና ወደዝህ ጤና ተቋም እንድታመጭዉ መጀመርያ የወሰነዉ ማን ነበር? | 1. እነ  2. አባቱ  3. አባቴ  4. ቅድሜ ኣያት  5. ሌላ ካሌ ይግለጹ | | | |  |  |
| 307 | ህጻንሽ ከዝህ በፊት ባለፉት 6 ወራት ዉስጥ በተቅማጥ ተይዞ ያዉቅ ነበር? | 1. አዎ  2. አያዉቅም | | | |  |  |
| 308 | ለጥያቄ ቁጥር 307፤ መልሱ አዎ ከሆኔ፤ ጤና ተቋም ጎብኝተሸ ነበር? | 1. አዎ  2. አይደለም | | | | መልሱ አይደለም ከሆኔ ወደ 312 ይለፉ፡፡ |  |
| 309 | ለጥያቄ ቁጥር 308፤ መልሱ አዎ ከሆኔ፤ ያኔ መምጣትሽ ለዝህ ጉብኝት ጠቅሞሽ ነበር? | 1. አዎ  2. አይደለም | | | |  |  |
| 310 | የጥያቄ ቁጥር 309 መልስ አዎ ከሆነ እንዴት? | 1. ለተቅማጥ ጤና ተቋም መጎብኘት ያለዉ ጠቀሜታ ላይ መክረዉኛል  2. ለተቅማጥ ጤና ተቋም አለመጎብኘት ያለዉ ጉዳት ነግረዉኛል  3. በፊት በተሰጠኝ ህክምና አገልግሎት ስለረካሁ  4. በፊት በተሰጠኝ ክብር ስለረካሁ  5. ለህጻኑ በተሰጠዉ ምርመራ ስለረካሁ  6. ሌላ ካሌ ይጥቀሱ | | | |  |  |
| 311 | ባለፌዉ በስድስት ወር ዉስጥ ባለዎት ጉብኝት ወደ ጤና ተቋም ከገቡበት እስከ ወጡበት ምን ያህል ሳዓት ነበር የቆዩት? | __________________(በደቂቃ) | | | |  |  |
| 312 | የጥያቄ ቁጥር 308 መልስ አይደለም ከሆኔ፤ህጻኑ እንዴት ነበር የዳነዉ? | 1. ወደ ባህላዊ ህክምና ወስጄ  2. እራሴ በቤት አክመ  3. መድኃኒትን ከፋርማስ/ ከመድኃኒት መደብሬ ያለ ሐክም ትዕዛዝ ገዝቸ  4. በቅዱስ ዉሐ አክመ  5. በራሱ ግዜ ተሸሎታል  6. ሌላ ካሌ ይጥቀሱ | | | |  |  |
| 312 | ከዝህ በፊት ከቤታችሁ/ከሰፌራችሁ በተቀማጥ ተይዞ የሞቴ ህጻን ነበሬ ወይ? | 1. አዎ  2. የለም | | | |  |  |
| **ክፍል አራት፡ -የህክምና አገልግሎት ለማግኘት ያለዉ ፍጥነት** | | | | | | | |
| 401 | ህጻንሽን ተቅማጥ ከጀመረዉ በኃላ ምን ያህል ግዜ ቆይተሸ ነዉ ወደ ህክምና ተቋም ያመጣሽዉ? | 1. ተቅማጥ ከጀመረዉ በ24 ሳኣት ዉስጥ  2. ተቅማጥ ከጀመረዉ ከ24-48 ሳዓት ዉስጥ  3. ተቅማጥ ከጀመረዉ 48-72 ሳዓት ዉስጥ  4. ተቅማጥ ከጀመረዉ 3-7 ቀን ዉስጥ  5. ተቅማጥ ከጀረዉ ከ7 ቀን በኃላ | | | |  |  |
| 402 | መልሱ ተቅማጥ ከጀመረዉ ከ 1 ቀን በኃላ ከሆነ፤ የህክምና አገልግሎት ተቅማጡ እንደጀመሬ ወድያዉ እንዳይፈልጉ ያደረጓቸዉ ምክንያቶች ምን ምን ነበሩ? | 1. በሽታዉ ከቆይታ በኃላ በራሱ ግዜ ስለምሻል  2. በሽታዉ በፊት በዝህ ህጻን ላይ/በሌላኛዉ ላይ በራሱ ግዜ ስለተሻለ  3. ትራንስፖርት አስቸጋር ስለሆኔ  4. የህክምና ወጪ ስለምያስቸግር  5. በጤና ባለሙያዎቹ እዉቀት ላይ እምነት ስለለለኝ  6. በጤና ተቋም ዉስጥ ወድያዉ ህክምና የማይሰጡ/ረዥም ሳዓት የምያስጠብቁ መስሎኝ ስለፈራሁ  7. ባህላዊ ህክምና በቤት ዉስጥ ስለምሰጥ  8. መድኃኒትን ከመድኃኒት መደበር ስለገዛሁ  9. ለህመሙ ህክምና ስለለለዉ  10. ግዜ ስለለለኝ  11. ገንዘብ ስለለለኝ  12. ህመሙ ቀላል ስለሆነ  13. ሌላ ካለ ይጥቀሱ | | | |  |  |
| 403 | ባህላዊ ህክምና መርጠዉ ከሆኔ፤ ያንን የመረጡበት ዋነኛ ምክንያት ምን ነበር? | 1. በህክምና እንክብካቤ ስለማይድን  2. ብዙ ብር ስለማይጠይቁ  3. ተገልጋይ አክባር ስለሆኑ  4. ረዥም ሳዓት ስለማያስጠብቁ  5. ህክምናቸዉ ዉጤታማ ስለሆነ  6. ምስጥረን ስለምጠብቁኝ  7. የግል መረጃዬ ስለምጠብቁ  8. ምክንያቱም ቤተሰቡ ይህንን መክሯታል  9. ምክንያቱም ቅርብ ስለሆኑ  10. ሌላ ካለ ይጥቀሱ | | | |  |  |
| 404 | የግል መድኃኒት አቅራቢዎች ከመረጡ፤ ምክንያቱ ምንድን ነው? | 1. ብዙ ዋጋ አያስከፍሉም  2. እነሱ ተገልጋይ አክባር ናቸው  3. ረዥም ሳዓት ስላማያስጠብቁ  4. ህክምናቸዉ ውጤታማ ስለሆነ  5. ምስጢራዊነትን ስለምጠብቁ  6. የግል መረጃን ስለምጠብቁ  7. ቤተሰቡ ይህንን መክሮታል  8. ምክንያቱም እነሱ ቅርብ ናቸው  9. ሌላ (ይግለጹ) ....... | | | |  |  |
| 405 | የህክምና አገልግሎት የፈለጉት በመጀመርያ ቀን ዉስጥ ከሆኔ፤ ወድያዉ የህክምና እንክካቤ እንድፈልጉ ያደረጓቸዉ ምክንያቶች ምን ምን ነበሩ? | 1. በፊት ሳልሔድ ቆይቸ በኃላ ላይ የበሽታዉ መጠን ስለከበደበት  2. ምልክቶቹ ከባድ ስለነበሩ  3. ቶሎ ስለመዉሰድ ጥቅም መረጃ ስላገኘሁ  4. ሌላ(ይግለጹ)_____________ | | | |  |  |
| 406 | ለህጻናት ተቅማጥ ህክምና ቶሎ ስለመዉሰድ ጥቅም መረጃ አግኝተዉ ከሆኔ፤ መረጃ ያገኙት ከዬት ነበር? | 1. ከጤና ባለሙያዎች  2. ከጤና ኤክስተንሽን ሠራተኞች  በስልጠና/በጤና ትምህርት  3. በመህበረሰብ ዉይይት ግዜ  4. ከጎሮቤት  5. ከሚድያ(ተለቭዥን፤ራድዮ)  6. ሌላ(ይግለጹ)_______________ | | | |  |  |
| **ክፍል አምስት: -ስለ መድሐኒት አገልግሎት የተገልጋዮች ዕይታ** | | | | | | | |
|  | | በጣም እስማማለሁ | እስማማለሁ | መካከለኛ ዕይታ | አልስማማም | በጣም አልስማማም |  |
| 501 | የፋርማስ ባለሙያዎች በህክምና አገልግሎትሽ ዉስጥ በጣም ጠቃም ናቸዉ፡፡ | 🞏 | 🞏 | 🞏 | 🞏 | 🞏 |  |
| 502 | የፋርማስ ባለሙያዎች የመድኃኒት አጠቃቀም ላይ አቅጣጫ ያሳያሉ | 🞏 | 🞏 | 🞏 | 🞏 | 🞏 |  |
| 503 | የፋርማስ ባለሙያዎች ስለመድኃኒት ጥቅም ምክር እና ትምህርት ይሰጣሉ | 🞏 | 🞏 | 🞏 | 🞏 | 🞏 |  |
| 504 | የፋርማስ ባለሙያዎች በህክምና አወሳሰድሽ ላይ ለምፌጠረዉ ችግር ጣልቃ ይገባሉ | 🞏 | 🞏 | 🞏 | 🞏 | 🞏 |  |
| 505 | የፋርማስ ባለሙያዎች የህክምና ምላሽሽን ይከታተላሉ | 🞏 | 🞏 | 🞏 | 🞏 | 🞏 |  |
| 506 | የፋርማስ ባለሙያዎች በሆስፕታል ፋርማስ ዉስጥ መድኃኒት የመስጠት ኃላፍነት አላቸዉ | 🞏 | 🞏 | 🞏 | 🞏 | 🞏 |  |
| 507 | የፋርማስ ባለሙያዎች የመድኃኒት ምርት ምርጫ ላይ በጣም ጥሩ ናቸዉ | 🞏 | 🞏 | 🞏 | 🞏 | 🞏 |  |
| 508 | የፋርማስ ባለሙያዎች በህክምና ጉዳዮች ላይ ምክር ለመስጠት ብቁ አይደሉም | 🞏 | 🞏 | 🞏 | 🞏 | 🞏 |  |
| 509 | የፋርማስ ባለሙያዎች የጤና ባለሙያዎች ናቸዉ | 🞏 | 🞏 | 🞏 | 🞏 | 🞏 |  |
| 510 | በጣም ከባድ ለሆኑ ለጤና ችግሮች የፋርማስ ባለሙያን ማወያየት ትችያለሽ | 🞏 | 🞏 | 🞏 | 🞏 | 🞏 |  |
| **ክፍል ስድስት፡ -የተገልጋዮች የፋርማስ አገልግሎት እርካታ መለክያ ጥያቄዎች** | | | | | | | |
|  |  | እጂግ በጣም ጥሩ | በጣም ጥሩ | ጥሩ | ተመጣጣኝ | ዝቅተኛ |  |
| 601 | የታዘዘልሽ መድኃኒት በፋርማስ ዉስጥ ተገኝቷል | 🞏 | 🞏 | 🞏 | 🞏 | 🞏 |  |
| 602 | የፋርማስ ባለሙያዎች የታዘዘልሽን መድሓኒት ስያመጡልሽ ጥንቃቄ ያደርጋሉ | 🞏 | 🞏 | 🞏 | 🞏 | 🞏 |  |
| 603 | ከፋርማስ ባለሙያ ጋር የምታዎርዉ በምስጥር ተጠብቋል | 🞏 | 🞏 | 🞏 | 🞏 | 🞏 |  |
| 604 | የፋርማስ ባለሙያዉ ጎንዮሽ ጉዳት ምን ያህል ገልጾልሻል | 🞏 | 🞏 | 🞏 | 🞏 | 🞏 |  |
| 605 | የመድኃኒት ዋጋ በፋርማስ ዉስጥ ተመጣጣኝ መሆኑ | 🞏 | 🞏 | 🞏 | 🞏 | 🞏 |  |
| 606 | የፋርማስ ባለሙያዎች በህክምና ጉዳይ ከእርሶ ጋር የምያሳልፉት ግዜ | 🞏 | 🞏 | 🞏 | 🞏 | 🞏 |  |
| 607 | የፋርማስ ባለሙያዎች በመድኃኒት አጠቃቀም ላይ የምሰጡት ትዕዛዝ ግልጸኝነት | 🞏 | 🞏 | 🞏 | 🞏 | 🞏 |  |
| 608 | የፋርማስ ባለሙያዎች ጥያቀሽን ምን ያህል ይመልሳሉ | 🞏 | 🞏 | 🞏 | 🞏 | 🞏 |  |
| 609 | የፋርማስ ባለሙያዎች መድኃኒት ከተጠቀሙ በኃላ ለምኖሬዉ ዉጤት የምሰጡት መረጃ | 🞏 | 🞏 | 🞏 | 🞏 | 🞏 |  |
| 610 | በመድኃኒት ማዘዣዉ ላይ ተሞልቶ እስክያልቅ ድረስ የምጠብቁት ሳዓት | 🞏 | 🞏 | 🞏 | 🞏 | 🞏 |  |
| 611 | የፋርማስ ባለሙያዉ የታዘዘልሽ መድሓኒት ትክክለኛነት ለማረጋገጥ ከዶክትርሽ ጋር የምሰራበት ሁኔታ | 🞏 | 🞏 | 🞏 | 🞏 | 🞏 |  |
| 612 | አጠቃላይ የፋርማስ አገልግሎትሽ ዉጤት | 🞏 | 🞏 | 🞏 | 🞏 | 🞏 |  |
| **ክፍል ሰባት፡ -የጤና ባለሙያዉ የተገልጋይ አክባርነት** | | | | | | | |
|  | | በጣም እስማማለሁ | እስማማለሁ | መካከለኛ | አልስማማም | በጣም አልስማማም |  |
| 701 | ባለሙያዉ ስያዋራኝ እንዴ ጓደኛ ነበር ያዋራኝ | 🞏 | 🞏 | 🞏 | 🞏 | 🞏 |  |
| 702 | ቤተስቦቸ በህክምና አሠጣጠ ላይ እንድሳተፉ እንደምፈልግና እንደማልፈልግ ጠይቆኛል | 🞏 | 🞏 | 🞏 | 🞏 | 🞏 |  |
| 703 | እነ ለራሰ እንክብካቤ ያለኝን ፍላጎት ሰምቶልኛል | 🞏 | 🞏 | 🞏 | 🞏 | 🞏 |  |
| 704 | በራሰ ህክምና እንክብካቤ ዉሳነ ላይ ረድቶኛል | 🞏 | 🞏 | 🞏 | 🞏 | 🞏 |  |
| 705 | በራሰ ህክምና እንክብካቤ ላይ ሀሳብ እንድሰጥ ረድቶኛል | 🞏 | 🞏 | 🞏 | 🞏 | 🞏 |  |

ስለሰጡን መረጃ ከልብ እናመሰግናለን!!
